# Assembly and dynamics of the bacteriophage T4 homologous recombination machinery

**DOI:** 10.1186/1743-422X-7-357

**Published:** 2010-12-03

**Authors:** Jie Liu, Scott W Morrical

**Affiliations:** 1 Section of Microbiology, University of California-Davis, Davis, CA 95616 USA; 2 Department of Biochemistry, University of Vermont College of Medicine, Burlington, VT 05405 USA

## Abstract

Homologous recombination (HR), a process involving the physical exchange of strands between homologous or nearly homologous DNA molecules, is critical for maintaining the genetic diversity and genome stability of species. Bacteriophage T4 is one of the classic systems for studies of homologous recombination. T4 uses HR for high-frequency genetic exchanges, for homology-directed DNA repair (HDR) processes including DNA double-strand break repair, and for the initiation of DNA replication (RDR). T4 recombination proteins are expressed at high levels during T4 infection in *E. coli*, and share strong sequence, structural, and/or functional conservation with their counterparts in cellular organisms. Biochemical studies of T4 recombination have provided key insights on DNA strand exchange mechanisms, on the structure and function of recombination proteins, and on the coordination of recombination and DNA synthesis activities during RDR and HDR. Recent years have seen the development of detailed biochemical models for the assembly and dynamics of presynaptic filaments in the T4 recombination system, for the atomic structure of T4 UvsX recombinase, and for the roles of DNA helicases in T4 recombination. The goal of this chapter is to review these recent advances and their implications for HR and HDR mechanisms in all organisms.

## Introduction

**Figure 1:**
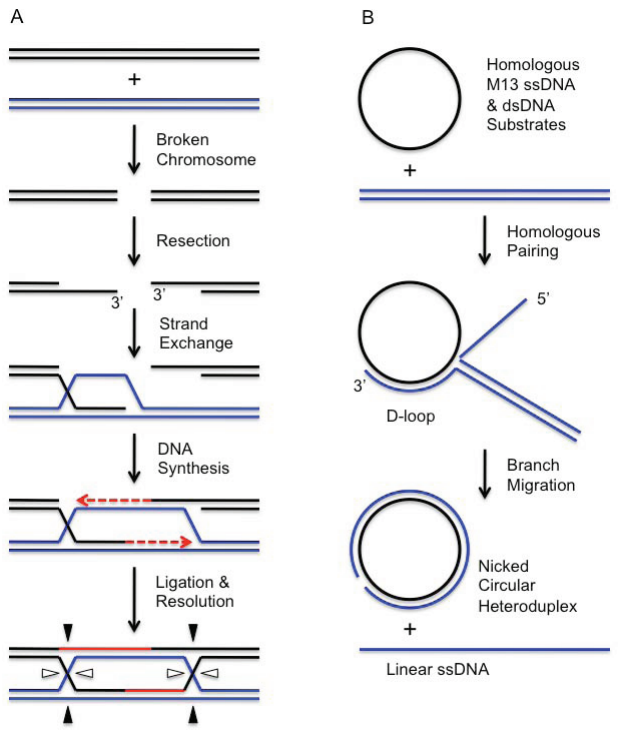
**DNA strand exchange assay and the role of DNA strand exchange in double-strand break repair.** Chromosome breakage is followed by nucleolytic resection to generate 3' ssDNA tails on the broken ends. The exposed ssDNA tails are the substrates for DNA strand exchange catalyzed by recombinases of the RecA/Rad51/UvsX family in collaboration with SSB, RMP, and other recombination proteins. The invasion of a homologous duplex (blue) by one of the 3' ssDNA tails generates a heteroduplex D-loop intermediate in which the 3' end of the invading strand is annealed to a template strand and can serve as a primer for recombination-dependent DNA replication (red). Strand displacement DNA synthesis in the forward direction (left to right as drawn) expands the D-loop until the displaced strand can anneal to the exposed ssDNA on the remaining DNA end. This 3' end can now prime DNA synthesis in the reverse direction (right to left as drawn). Ligation generates Holliday junctions that can branch migrate and ultimately are resolved by structure-specific endonucleses to generate recombinant products (not shown). (B) Classic in vitro assay for DNA strand exchange activity of RecA/Rads51/UvsX family recombinases. Homologous circular ssDNA and linear dsDNA substrates derived from bacteriophage M13 are incubated with recombinase and accessory proteins in the presence of ATP. Recombinase-catalyzed homologous pairing generates partially heteroduplex D-loop intermediates. Polar branch migration driven by the recombinase and/or helicases extends the heteroduplex to generated nicked circular dsDNA and linear ssDNA products.

Homologous recombination (HR) is a conserved biological process in which DNA strands are physically exchanged between DNA molecules of identical or nearly identical sequence (Figure 1). The DNA strand exchange mechanism in HR allows gene conversion events to occur, which is important for maintaining genetic diversity within populations of organisms. The DNA strand exchange mechanism in HR is also essential for the high-fidelity repair of DNA double-strand breaks (DSBs) and daughter-strand gaps, which is important for maintaining genome stability [1-3]. These homology-directed DNA repair (HDR) processes require the coordination of activities between HR and DNA replication machineries.

### Homologous recombination in bacteriophage T4

The bacteriophage T4 recombination system provides an important model for understanding recombination transactions including DNA strand exchange, recombination-dependent replication (RDR), and homology-directed DNA repair (HDR) [4-6]. The relatively simple, but functionally conserved, core recombination machinery of T4 facilitates detailed mechanistic studies of DNA strand exchange reactions and intermediates. The T4 paradigm for presynaptic filament assembly is widely used as a basis for studying presynaptic filaments in many cellular organisms including humans. At the same time, because of the close linkages between its DNA recombination, replication, and repair pathways, bacteriophage T4 has yielded novel insights on the cross-talk that occurs between recombination and replication proteins. This is especially true in the case of T4 DNA helicases, which are seen as critical for the channeling of recombination intermediates into RDR and HDR pathways.

### Single-stranded DNA and presynaptic filaments

**Figure 2:**
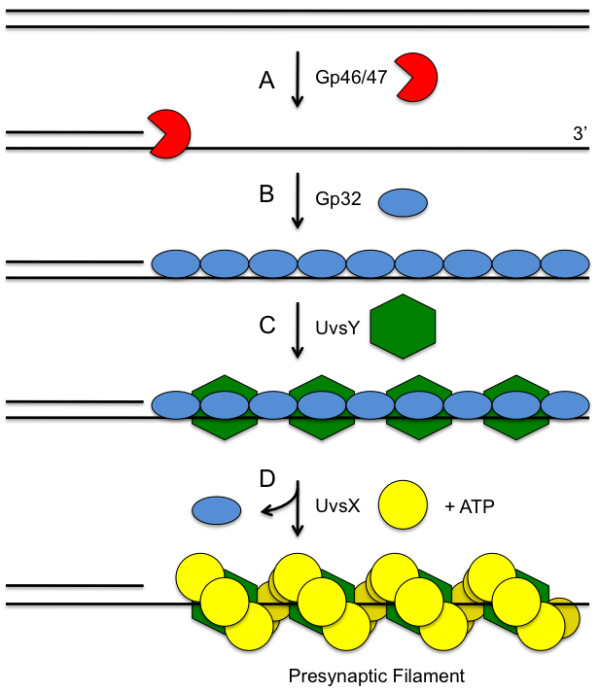
**Presynapsis pathway in bacteriophage T4 homologous recombination**. (A) A dsDNA end may be nucleolytically resected to expose a 3' ssDNA tail. The Gp46 and Gp47 proteins are thought to be the major enzymes involved in the resection step. (B) The exposed ssDNA is sequestered by the Gp32 ssDNA-binding protein, which denatures secondary structure in ssDNA and keeps it in an extended conformation. (C) The UvsY recombination mediator protein forms a tripartite complex with Gp32 and ssDNA and "primes" the complex for recruitment of UvsX recombinase. (D) UvsY recruits ATP-bound UvsX protein and nucleates presynaptic filament formation. Gp32 is displaced in the process.

The generation of single-stranded DNA is a common early step of HR pathways [7,8]. ssDNA production typically occurs as a result of nucleolytic resection of DSBs (Figure 1), or due to replication fork stalling or collapse. In T4 recombination, exonuclease activities of a Gp46/Gp47 complex (orthologous to eukaryotic Mre11/Rad50) appear to be critical for DSB resection [9]. In addition to DNA damage-linked production of ssDNA, bacteriophage T4 routinely generates ssDNA during replication of its linear chromosome ends. The production of ssDNA tails or gaps in otherwise duplex DNA allows the assembly of core recombination machinery including *presynaptic filaments *on ssDNA. Presynaptic filaments are helical nucleoprotein filaments consisting of a recombinase enzyme and its accessory proteins bound cooperatively to ssDNA (Figure 2). Presynaptic filament assembly activates the enzymatic activities of the recombinase including ATPase and DNA strand exchange activities. Filament dynamics controls DNA strand exchange and its coupling to the downstream, replicative steps of HDR. These processes require the timely assembly of presynaptic filaments on recombinagenic ssDNA. Equally important is the coordinated disassembly or translocation of filaments, which appears to be required to make way for the assembly of replication enzymes on recombination intermediates [10,11].

### The transition from recombination to DNA replication and repair

The transition from recombination intermediate to replication fork occurs very efficiently in bacteriophage T4, which has evolved to use this as a major mode of DNA replication initiation. The transition likely involves not only the built-in dynamics of the presynaptic filament but also the coordinated activities of DNA helicases. In the following sections of this chapter, we will review what is known about presynaptic filament dynamics in the T4 system, as well as what is known about the influences of DNA helicases on recombination, and how these two ATP-driven machines may cooperate with each other to successfully couple HR to recombination-dependent replication and repair.

## Properties of the T4 Core Recombination Machinery

Although relatively simple, the core activities of the T4 recombination system are highly conserved. Three core protein components are required for T4 presynaptic filament assembly and for DNA strand exchange under physiological conditions: UvsX, the phage recombinase (orthologous to bacterial RecA and eukaryotic Rad51); Gp32, the phage ssDNA-binding protein (equivalent to bacterial SSB and eukaryotic RPA); and UvsY, the phage recombination mediator protein (equivalent to bacterial RecOR, eukaryotic Rad52, Brca2, and others) [4,5]. The DNA binding properties of UvsX, Gp32, and UvsY are presented below in context with their physical and enzymatic properties.

### UvsX recombinase

UvsX protein (44 kDa) is a member of the RecA/Rad51 recombinase family and shares 28% sequence identity and 51% sequence similarity with the catalytic core domain of *E. coli *RecA [12]. UvsX catalyzes DNA strand exchange reactions that play central roles in T4 HR, RDR, and HDR pathways [4,6]. UvsX binds sequence-non-specifically to both ssDNA and dsDNA and can bind to both lattices simultaneously via two different binding sites (Maher, R.L. and S.W. Morrical: Coordinated binding of ssDNA and dsDNA substrates by UvsX recombinase and its regulation by ATP, unpublished). UvsX has higher affinity for dsDNA in the absence of other factors, but simultaneous ssDNA binding lowers UvsX-dsDNA binding affinity unless the duplex sequence is homologous to the bound ssDNA (Maher, R.L. and S.W. Morrical: Coordinated binding of ssDNA and dsDNA substrates by UvsX recombinase and its regulation by ATP, unpublished). At the same time, UvsX-ssDNA interactions are selectively stabilized by nucleoside triphosphates ATP, dATP, or their non-hydrolyzable analogs, and by UvsY protein [13,14]. These combined factors help to target UvsX filament assembly onto recombinagenic ssDNA even in the presence of excess dsDNA as would normally be found in the T4-infected cell. Binding of UvsX to ssDNA, not dsDNA, specifically activates catalysis by UvsX including ATPase and DNA strand exchange activities.

Quantitative binding studies established the intrinsic ssDNA-binding parameters of UvsX [13]. Its average binding site size on ssDNA is 4 nucleotide residues per protomer. UvsX exhibits moderate affinity and cooperativity for ssDNA with *K*_obs _= *K*ω ≈ 10^6 ^M^-1 ^at physiological ionic strength, where the cooperativity parameter ω ≈ 100 [13]. The observed cooperativity of UvsX is consistent with the formation of long filaments on ssDNA at high binding density.

The ATPase activity of UvsX is strongly ssDNA-dependent under normal solution conditions [15], although very high salt concentrations can also stimulate ATP hydrolysis by UvsX in the absence of ssDNA. Double-stranded DNA does not activate UvsX ATPase activity. UvsX ATPase activity is also highly unusual in that it generates both ADP and AMP as products [15,16]. The two products appear to be generated independently by two different classes of active sites within UvsX-ssDNA presynaptic filaments, as indicated by results of steady-state kinetics studies [16]. These sites have different *K*_m _and *k*_cat_/*K*_m _values for the ATP and ssDNA substrates. One type of active site appears to produce ADP exclusively, while the other appears to generate AMP via a sequential mechanism (ATP → ADP → AMP) without releasing the ADP intermediate from the active site [16]. Thus UvsX presynaptic filaments exhibit active site asymmetry (Figure 2). This asymmetry may be important for UvsX-catalyzed DNA strand exchange reactions, since increases in ADP/AMP product ratio observed in UvsX site-directed mutants correlate inversely with strand exchange activity [16]. Active site asymmetry may be a general property of presynaptic filaments in many species, since evidence exists for two classes of active sites in filaments of *E. coli *RecA and *S. cerevisiae *Rad51 recombinases [17,18].

UvsX-ssDNA filaments rapidly search for homology in dsDNA substrates, leading to efficient homologous pairing and strand exchange. ATP binding (not hydrolysis) is required for homologous pairing, however ATP hydrolysis is needed to drive extensive polar (5' → 3') branch migration during strand exchange [19-21]. There is a strong requirement for Gp32 to stimulate UvsX-catalyzed strand exchange at normal concentrations of the recombinase [15,22,23]. In vitro, this Gp32 requirement can be circumvented by raising the UvsX concentration to super-saturating levels with respect to ssDNA binding sites. Stimulation of strand exchange by Gp32 requires the correct order of protein addition: Adding Gp32 to ssDNA prior to the addition of UvsX typically inhibits strand exchange. This ssDNA-binding protein/recombinase order of addition effect is a characteristic of all well-characterized recombination systems [24], and is reflective of the competition between the two proteins for binding sites on ssDNA. Similar inhibition of UvsX-catalyzed strand exchange is seen at high concentrations of Gp32 and/or at elevated salt concentrations, i.e. conditions that favor Gp32-ssDNA over UvsX-ssDNA interactions. Under conditions such as these there is an absolute requirement for the UvsY recombination mediator protein for strand exchange reactions in vitro [23,25]. This mimics the in vivo situation in which T4 recombination transactions are equally dependent on UvsX and UvsY [26-28].

Branched networks of single- and double-stranded DNA are the major products of UvsX-catalyzed DNA strand exchange, indicating that each DNA substrate molecule participates in many homologous pairing events [15,29]. One plausible explanation for this behavior is that UvsX appears to catalyze homologous pairing much more rapidly than branch migration. Therefore it is possible for different regions of one long ssDNA substrate to pair with homologous regions of different dsDNA substrates before any of the resulting D-loop intermediates can be completely extended into heteroduplex DNA. Rapid homologous pairing by UvsX may be an evolutionary adaptation for efficiently capturing 3' ssDNA tails and using them to prime recombination-dependent replication. Furthermore, branch migration appears to be dependent on T4-encoded DNA helicases, as we discuss in a later section.

### Gp32 ssDNA-binding protein

Gp32 (34 kDa) is the prototype ssDNA-binding protein and a key component of the T4 replisome. Gp32 also plays important roles in homologous recombination and DNA repair. The biochemical properties of Gp32 have been thoroughly characterized [30-45], and the atomic structure of its central DNA-binding domain (DBD) has been solved [32]. The DBD contains an oligonucleotide/oligosaccharide-binding (OB)-fold motif plus a structural Zn^++ ^atom. An N-terminal domain (so-called *basic *or "B-domain") is required for self-association and cooperativity, whereas a C-terminal domain (so-called *acidic *or "A-domain") is the site for protein-protein interactions with various recombination and replication enzymes including UvsX and UvsY.

Gp32 binds sequence-non-specifically to polynucleotides, with the highest observed affinity for ssDNA (*K*_obs _≈ 10^9 ^M^-1 ^at physiological ionic strength), moderate affinity for single-stranded RNA, and very low affinity for dsDNA. The binding site size of Gp32 on ssDNA is approximately 7 nucleotide residues. Binding to ssDNA is highly cooperative (ω ≈ 1000), meaning that Gp32 exists almost exclusively in clusters or long filaments on ssDNA at protein concentrations normally encountered in in vitro DNA strand exchange assays as well as in vivo.

Gp32 affects both pre- and post-synaptic steps of UvsX-catalyzed DNA strand exchange reactions [15,22,23,25,46,47]. An important function of Gp32 in presynapsis is to denature secondary structure in the ssDNA substrate, which *eventually *allows UvsX to saturate the ssDNA by forming long presynaptic filaments. Paradoxically, the immediate effect of Gp32 on UvsX-ssDNA filament formation is negative under physiological conditions, because Gp32 competes effectively with UvsX for binding sites [13]. Overcoming Gp32 inhibition requires either pre-incubation of UvsX with ssDNA in the presence of ATP (the previously mentioned order of addition effect), or the inclusion of UvsY in reaction mixtures (see below) [4,24]. Gp32 has also been shown to play a post-synaptic role in strand exchange, stimulating the reaction by sequestering the outgoing ssDNA strand that is displaced during D-loop formation and subsequent branch migration [47].

### UvsY recombination mediator protein

UvsY is the prototype recombination mediator protein or RMP [24]. By definition, RMPs are proteins that load recombinases of the RecA/Rad51 family onto ssDNA molecules that are pre-saturated with cognate ssDNA-binding protein. UvsY is absolutely required for UvsX-catalyzed DNA strand exchange in the presence of Gp32 under physiological or high-salt conditions [22,48,49]. In vivo, UvsY is also absolutely required for UvsX-dependent recombination since mutations knocking out either gene product have equivalent recombination-deficient phenotypes including the small-plaque phenotype associated with defective RDR [26-28]. UvsY is the only member of the core T4 recombination machinery that forms a discreet oligomeric structure: It exists as a stable hexamer of identical 15.8 kDa subunits in solution, and binds to ssDNA in this form [50].

UvsY binds to both ssDNA and dsDNA, but has a much higher affinity for the former under relaxed DNA conditions [51]. The preference of UvsY for ssDNA may be an important factor in directing UvsX filament assembly onto ssDNA in the presence of excess dsDNA, since UvsX itself has a relatively high affinity for non-homologous dsDNA (Maher, R.L. and S.W. Morrical: Coordinated binding of ssDNA and dsDNA substrates by UvsX recombinase and its regulation by ATP, unpublished). UvsY has a binding site size on ssDNA of 4 nucleotide residues per protomer, or 24 nucleotide residues per hexamer [52]. The protomeric binding site sizes of UvsY and UvsX are identical. UvsY binds to ssDNA with high affinity (*K*-_obs _≈ 10^7 ^M^-1 ^at physiological ionic strength), but with little or no cooperativity (ω ≈ 1). Therefore UvsY has higher *intrinsic *affinity, but lower cooperativity, for ssDNA than either UvsX or Gp32 under conditions that are relevant for strand exchange reactions in vitro and in vivo. UvsY-ssDNA interactions are weakened by mutations at residues Lys-58 and Arg-60, which form part of a conserved LKARLDY motif (so-called 'KARL' motif) found in the N-terminal domain of UvsY, which is thought to comprise part of its DNA binding surface [14,48,51,53,54]. The KARL motif is also found in certain DNA helicases, however no helicase activity has ever been associated with UvsY, which lacks a motor domain. The C-terminal domain of UvsY is essential for hexamerization. Deletion of this domain drastically reduces the affinity of UvsY-ssDNA interactions, demonstrating the importance of UvsY hexamers as the relevant ssDNA-binding unit [55].

Several lines of evidence indicate that UvsY hexamers have the ability to wrap ssDNA strands around themselves, and that wrapping is responsible for the high affinity of UvsY-ssDNA interactions. Evidence includes the observation that a C-terminally deleted, monomeric form of UvsY has 10^4^-fold lower affinity for ssDNA than wild-type [55]. The wrapping hypothesis is supported by the finding that mutiple subunits within each UvsY hexamer are in contact with ssDNA [51]. Other evidence comes from results of single-molecule DNA stretching studies, which showed that the ssDNA that is created by the treatment of individual stretched dsDNA molecules with glyoxal is strongly wrapped by UvsY [54]. Wrapping of ssDNA occurs at low stretching forces where the DNA is relatively relaxed. At high stretching forces, where the DNA is under tension, wrapping is suppressed. The tension-dependent suppression of wrapping leads to the loss of preferential binding to ssDNA as shown by the fact that UvsY binds tighter to stretched dsDNA than to stretched ssDNA [54]. This contrasts with the observation that UvsY has ~1000-fold higher affinity for ssDNA than for dsDNA under relaxed conditions [51]. Therefore high-affinity binding of UvsY to ssDNA requires wrapping, which also imposes a preference for binding to ssDNA over dsDNA. Presumably UvsY cannot wrap dsDNA because its persistence length is much higher than that of ssDNA [56]. The surprising observation that UvsY binds tightly to stretched dsDNA could have important implications for presynaptic filament assembly. The binding of Gp32 to ssDNA creates an extended or "stiff" DNA conformation that might be recognized by UvsY in an unwrapped mode similar to its interaction with stretched dsDNA. Converting this extended ssDNA structure into a wrapped one might be an important step in the recruitment of UvsX recombinase, as we discuss in a later section.

UvsY is absolutely required for UvsX-catalyzed DNA strand exchange assays performed under physiological conditions of Gp32 and salt [4,24], consistent with the co-dependency of recombination on UvsX and UvsY in vivo [26-28]. In vitro, UvsY lowers the critical concentration of UvsX for RDR and other recombination reactions [46,57]. UvsY stimulates the ssDNA-dependent ATPase activity of UvsX, possibly by acting as a nucleotide exchange factor for the recombinase [58]. The greatest stimulation of ATPase activity is seen when UvsY and Gp32 act together synergistically on the reaction [23,49]. UvsY stimulates the catalytic activities of UvsX mainly by promoting presynaptic filament assembly. The mechanism of UvsY's recombination mediator activity will be explored in greater detail below.

## Assembly and Dynamics of the T4 Presynaptic Filament

### Regulation of UvsX-ssDNA interactions by the ATPase cycle

Like all RecA/Rad51 recombinases, UvsX is a member of the AAA^+ ^ATPase super-family and its interactions with ssDNA are regulated by ATP binding and hydrolysis. The analog ATPγS, which is tightly bound but slowly hydrolyzed by UvsX, induces a stable, high-affinity ssDNA binding state of the enzyme [13,14]. ATP itself transiently induces high-affinity ssDNA binding by UvsX until it is hydrolyzed to ADP or AMP [15,16]. Both of these hydrolytic products are associated with decreased ssDNA-binding affinity states of UvsX under steady-state conditions [16].

### Regulation of protein-ssDNA interactions by UvsY

Most evidence indicates that UvsX and Gp32 undergo mutually exclusive binding to ssDNA [48,59,60]. On the other hand there is overwhelming evidence that UvsY can co-occupy ssDNA binding sites simultaneously with either UvsX or Gp32 [14,19,25,60-62]. The interaction of UvsY with either Gp32-ssDNA or UvsX-ssDNA complexes alters the properties of both in ways that favor presynaptic filament formation and the activation of UvsX catalytic activities.

UvsY forms a stable tripartite complex with Gp32 and ssDNA at physiologically relevant salt conditions [61]. These complexes contain stoichiometric amounts of both UvsY and Gp32 with respect to their normal binding site sizes on ssDNA (Figure 2). Gp32-ssDNA interactions are destabilized within the UvsY-Gp32-ssDNA complex as shown by their increased sensitivity to disruption by salt compared to Gp32-ssDNA complexes in the absence of UvsY [61]. Results of single-molecule DNA stretching studies confirm that UvsY destabilizes Gp32-DNA interactions [54]. It has been proposed that, since cooperativity is such a large component of *K*_obs _for Gp32-ssDNA interactions, UvsY could destabilize Gp32-ssDNA by lowering Gp32's cooperativity parameter [61]. This is probably the major pathway for destabilizing Gp32-ssDNA under physiological or high-salt conditions. It has also been proposed, based on results of single-molecule DNA stretching experiments, that UvsY directly displaces Gp32 from ssDNA under low-salt conditions [54]. In either case, the destabilization of Gp32-ssDNA interactions by UvsY lowers the energy barrier necessary for UvsX to displace Gp32 from ssDNA, which is necessary for nucleation and propagation of presynaptic filaments on ssDNA that is pre-saturated with Gp32 (as is likely to be the case in vivo).

Biochemical studies demonstrate that UvsY stabilizes UvsX-ssDNA interactions [14]. UvsY, UvsX, and ssDNA form a tripartite complex with a stoichiometry of ~1 UvsY hexamer per 6 UvsX protomers, consistent with their equivalent binding site sizes (4 nucleotide residues/protomer). The increased stability of UvsX-ssDNA interactions within these complexes is demonstrated by their higher resistance to salt compared to filaments formed in the absence of UvsY. The most stable complex is formed when UvsY and ATPγS are both present, indicating that the RMP and nucleoside triphosphate act synergistically to stabilize UvsX-ssDNA [14]. UvsY also stabilizes UvsX-ssDNA in the presence of ADP or no nucleotide, so its effects are global. Results of recent kinetics studies are consistent with the idea that UvsY acts as a nucleotide exchange factor for UvsX, promoting the release of hydrolytic products so that new ATP substrate can bind to the active sites [58]. It is postulated that UvsY-enhanced nucleotide exchange allows UvsX to remain longer in its ATP-bound form with higher affinity for ssDNA, which would tend to stabilize presynaptic filaments and increase their catalytic activites activity. Through its dual activities in destabilizing Gp32-ssDNA and stabilizing UvsX-ssDNA interactions, UvsY allows UvsX filaments to nucleate and propagate on Gp32-covered ssDNA (Figure 2).

### ssDNA hand-offs govern filament assembly

UvsX and UvsY interact specifically with the C-terminal "A-domain" of Gp32, and with each other [35,36,49,60]. Protein-protein interactions play a significant role in the overall DNA strand exchange reaction. Nevertheless, studies of UvsY have shown that its ability to destabilize Gp32-ssDNA complexes is independent of UvsY-Gp32 interactions [54,61], indicating that the ssDNA-binding activity of UvsY is responsible for destabilizing Gp32-ssDNA interactions. Results of in vitro complementation assays between UvsX and UvsY mutants further suggest that UvsY-ssDNA interactions create an optimal ssDNA conformation for high-affinity binding by UvsX [58]. Studies showed that UvsY KARL-motif mutants K58A and K58A/R60A have reduced affinities for ssDNA compared to wild-type [53]. Similarly UvsX missense mutants H195Q and H195A exhibit reduced affinities for ssDNA as well as altered enzymatic activities compared to wild-type [16]. Unlike wild-type UvsX, the ssDNA-dependent ATPase activities of UvsX-H195Q/A are strongly inhibited by wild-type UvsY at both low and high concentrations of the mediator. The UvsY KARL-motif mutants partially relieve this inhibition [58]. Furthermore the UvsX-H195Q mutant has weak DNA strand exchange activity that is inhibited by wild-type UvsY, but stimulated by the UvsY KARL-motif mutants [58]. These and other results support a mechanism in which presynaptic filament assembly involves a hand-off of ssDNA from UvsY to UvsX, with the efficiency of the hand-off controlled by the relative ssDNA-binding affinities of the two proteins.

**Figure 3:**
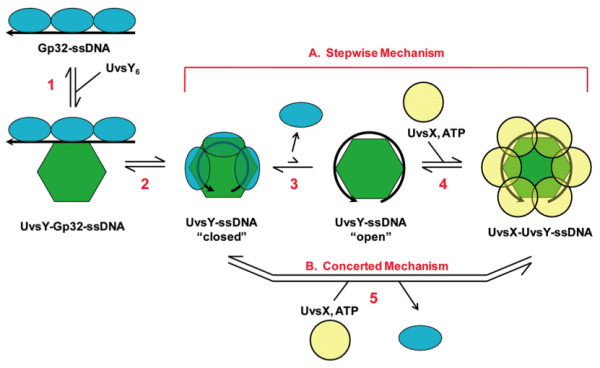
**UvsY promotes presynaptic filament assembly on Gp32-covered ssDNA by a double hand-off mechanism (adapted from **[51]). UvsY protein facilitates the loading of UvsX recombinase onto ssDNA and the concomitant displacement of Gp32 ssDNA-binding protein from ssDNA. The figure shows UvsX loading and Gp32 displacement from the perspective of a single UvsY hexamer, as if looking down the helical axis of a nascent presynaptic filament. The cooperative binding of Gp32 to ssDNA extends the polynucleotide lattice. The first handoff occurs as hexameric UvsY recognizes and binds to the extended ssDNA (Step 1), then converts it into a wrapped conformation(s) (Steps 2-3), destabilizing Gp32-ssDNA interactions in the process. The UvsY-wrapped ssDNA complex is postulated to be in equilibrium between "closed" and "open" conformations (Step 3), the latter of which is recognized by the ATP-bound form of UvsX protein to nucleate presynaptic filament assembly (Step 4) while displacing Gp32. (A) Steps 3-4 constitute a step-wise mechanism for Gp32 displacement and UvsX loading by UvsY, which may occur under low-salt conditions. (B) Under high-salt conditions UvsY does not displace Gp32 from ssDNA directly, so filament assembly likely occurs by a concerted mechanism in which synergistic action of UvsY and ATP-bound UvsX is required to displace Gp32.

Evidence increasingly supports the notion that DNA and RNA pathways channel their substrates through series of hand-off transactions in which intermediate nucleic acid structures are passed directly from one protein in the pathway to the next [63]. This strategy avoids potential cytotoxic effects of the free nucleic acid structure and protects it from unprogrammed side reactions or degradation. The available data suggest that T4 presynaptic filament assembly is also governed by a sequence of hand-off events involving intermediate ssDNA structures generated by Gp32 and UvsY (Figure 3). Initially, Gp32 binding converts ssDNA into an extended conformation that resembles the mechanically stretched DNA created in force-spectroscopy experiments. In the first hand-off event, a UvsY hexamer binds to the extended ssDNA and converts it into a wrapped conformation that destabilizes Gp32-ssDNA interactions. The wrapped UvsY-ssDNA complex is thought to be in equilibrium between "closed" and "open" states. The "closed" state destabilizes Gp32-ssDNA interactions but is inaccessible to UvsX, whereas the "open" state favors high-affinity UvsX-ssDNA interactions. In the second hand-off event, ATP-bound UvsX binds to the "open" form of the wrapped UvsY-ssDNA structure, allowing nucleation of a UvsX-ssDNA filament while displacing Gp32 from the ssDNA. Other ssDNA hand-off transactions may occur as the filament transitions from the nucleation to the propagation phase, or as UvsY performs its nucleotide exchange factor function. In addition, the linkage of the UvsX ATPase cycle to the sequential hand-off mechanism creates opportunities for dynamic instability in presynaptic filaments, which we will address in a later section.

### UvsX-Gp32 exchanges on ssDNA

Gp32F is a fluorescein-conjugated form of Gp32 that is useful as a fluorescence probe for Gp32 displacement from ssDNA and to study the kinetics of presynaptic filament assembly in real time [48]. As UvsX filaments assemble on Gp32F-covered ssDNA, Gp32F is displaced and the fluorescence of its fluorescein moiety decreases. This assay was used to study presynaptic filament assembly both in the absence of UvsY (low-salt conditions only) and in the presence of UvsY (physiological or high-salt conditions). The salt-dependence of the UvsY requirement for Gp32 displacement is a consequence of differential salt effects on the intrinsic association constants (*K *parameters) of UvsX and Gp32 for ssDNA [13,41,44,45,64]. Under low-salt conditions (≤ 50 mM NaCl), the ATP or ATPγS-bound forms of UvsX possess sufficient affinity for ssDNA to compete with Gp32 and displace it from the lattice, causing a time-dependent decrease in the fluorescence of the Gp32F probe [48]. ADP-bound, AMP-bound, or *apo *forms of UvsX cannot displace Gp32 from ssDNA under any conditions. At higher, more physiologically relevant salt concentrations, all forms of UvsX lack the ability to displace Gp32 from the ssDNA. Under these conditions, the addition of UvsY restores UvsX-ssDNA filament formation and Gp32 displacement, as measured by the decrease in Gp32F fluorescence [48]. The UvsY-dependent reactions still require ATP or ATPγS as a prerequisite for filament assembly; ADP-, AMP-, and *apo*-UvsX conditions do not support Gp32 displacement. This observation is consistent with the previous finding that UvsY and ATPγS-binding stabilize UvsX-ssDNA filaments synergistically [14], which implies the cooperation of these two factors during filament nucleation and/or propagation steps.

Following timecourses of Gp32F displacement from ssDNA allows detailed analyses of the kinetics of presynaptic filament assembly in a fully-reconstituted *in vitro *T4 recombination system (UvsX, UvsY, and Gp32). This has led to important new discoveries about filament dynamics and about the mechanism of UvsY in recombination mediation (Liu, J., C. Berger, and S.W. Morrical: Kinetics of Presynaptic Filament Assembly in the Presence of SSB and Mediator Proteins, unpublished). Under low-salt conditions, the ATP-dependent, UvsY-independent nucleation of UvsX filaments on Gp32F-covered ssDNA is highly salt-sensitive. Nevertheless nucleation rates are faster than propagation rates, suggesting that UvsX nucleates rapidly at many different sites. Under high-salt conditions, UvsY appears to specifically enhance the nucleation step to overcome the salt-sensitivity of UvsX filament assembly (Liu, J., C. Berger, and S.W. Morrical: Kinetics of Presynaptic Filament Assembly in the Presence of SSB and Mediator Proteins, unpublished). Rapid, salt-sensitive nucleation may be a general property of recombinase-DNA interactions, since similar behavior is observed for human Rad51 filament assembly on dsDNA [65]. It will be interesting to learn whether human RMPs such as Rad52, Brca2, or Rad51 paralogs also work by decreasing the salt-sensitivity of Rad51 filament nucleation.

**Figure 4:**
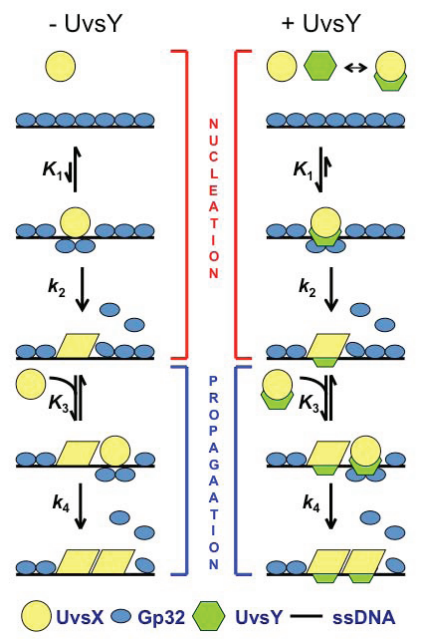
**Model for the kinetics of T4 presynaptic filament formation in the presence and absence of UvsY (adapted from Liu, J., C. Berger, and S.W. Morrical: Kinetics of Presynaptic Filament Assembly in the Presence of SSB and Mediator Proteins, unpublished) **. *Left -- *Under low-salt conditions in the absence of mediator protein UvsY, ATP-bound UvsX, a high affinity form, binds Gp32-ssDNA rapidly to form an unstable nucleation site or "pre-nucleation complex" (association constant *K*_1_). A slow but almost irreversible conformational change (forward rate constant *k*_2_) is required by UvsX to displace Gp32 and to secure this isolated nucleation site on the lattice. With successful nucleation, more ATP-bound UvsX is recruited to form an unstable cluster (association constant *K*_3_). This rapidly formed UvsX cluster undergoes another slow but almost irreversible conformational change to displace Gp32 and to redistribute into a stable and productive presynaptic filament (forward rate constant *k*_4_). *Right -- *Under high-salt conditions the mediator protein, UvsY, facilitates filament nucleation by stabilizing the salt-sensitive pre-nucleation complex (enhanced *K*_1_), by forming a special quaternary complex with UvsX, Gp32, and ssDNA. Filament propagation (particularly *k*_4_) is rate-limiting under all conditions.

A simplified kinetic scheme for T4 presynaptic filament assembly is shown in Figure 4, based on data derived from analysis of Gp32F displacement timecourses (Liu, J., C. Berger, and S.W. Morrical: Kinetics of Presynaptic Filament Assembly in the Presence of SSB and Mediator Proteins, unpublished). Results are consistent with a two-phase model, nucleation and propagation, both of which include a fast and reversible binding step (*K*_1 _or *K*_3_) followed by a slow isomerization step (*k*_2 _or *k*_4_) that is essentially irreversible under pre-steady-state conditions. We found that UvsY specifically enhances *K*_1_, thereby stabilizing the product of the reversible binding step during the filament nucleation phase. This product may be thought of as a "pre-nucleation complex". Therefore UvsY overcomes the salt-sensitivity of filament nucleation by stabilizing the pre-nucleation complex at high salt concentrations. We also found that *k*_4_, the rate constant for the isomerization step of filament propagation, is rate-limiting under all conditions (Liu, J., C. Berger, and S.W. Morrical: Kinetics of Presynaptic Filament Assembly in the Presence of SSB and Mediator Proteins, unpublished). This suggests that long presynaptic filaments are likely to be assembled from many shorter filaments that arise at multiple nucleation centers. In accord with this idea, human Rad51 assembles on dsDNA from many rapidly-formed nucleation sites and the cluster growth from each site is limited in length [65]. The requirement for many filament nucleation events may explain the observation that an apparent 1:1 stoichiometry between UvsX and UvsY has to be maintained for optimal recombination activity [22,46,60].

### Dynamic instability in presynaptic filaments

**Figure 5:**
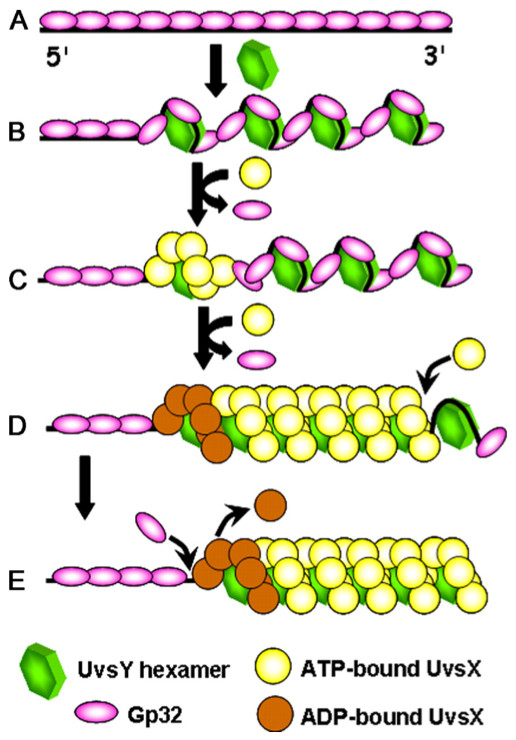
**Dynamic instability in T4 presynaptic filaments is coupled to the UvsX ATPase cycle and to UvsX/Gp32 competition for binding sites (adapted from **[48]**)**. A. Gp32 covers free ssDNA rapidly to protect it from nuclease digestion and to remove secondary structure. B. Hexameric UvsY protein weakens Gp32-ssDNA interactions by binding to the complex and wrapping the ssDNA lattice. C. ATP-bound UvsX is recruited to the tripartite UvsY-Gp32-ssDNA intermediate. ATP and UvsY both contribute to a synergistic increase in UvsX-ssDNA binding affinity that allows the recombinase to locally displace Gp32 from the lattice. D. Propagation occurs in the 5' → 3' direction as ATP-bound UvsX subunits slowly add to the 3' filament end, displacing more Gp32 subunits in the process. E. The first UvsX subunits to bind are the first to hydrolyze ATP, generating a relatively aged, ADP-capped 5' filament end. The ADP-bound UvsX subunits are now vulnerable to displacement by Gp32. Differential competitive effects between Gp32 and the ATP- vs. ADP-capped filament ends creates dynamic instability in the complex, which could lead to filament treadmilling.

Presynaptic filaments are predicted to exhibit dynamic instability, or vectorial growth and collapse, due to the coupling of the recombinase ATPase cycle to changes in ssDNA binding affinity [15,19,47,60]. The Gp32F probe provides an indirect readout of the dynamic instability of UvsX-ssDNA filaments [49]. Results demonstrate that the dynamic instability of T4 presynaptic filaments depends not only on UvsX-catalyzed ATP hydrolysis, but also on competition between UvsX and Gp32 for binding sites on ssDNA (Figure 5). Experiments were designed in which UvsX and Gp32 undergo a pre-steady-state competition for a limited number of binding sites on ssDNA at physiological ionic strength [48]. The order of addition is controlled so that ssDNA is added to a pre-existing mixture of recombination proteins, which mimics the most likely pathway for filament assembly/disassembly in vivo. Filament assembly/disassembly is then monitored by following Gp32F dissociation/association using fluorescence. The data show that presynaptic filaments formed in the presence of Gp32 undergo constant assembly and collapse that is closely linked to the ATPase cycle of UvsX [48]. The reactions occur in three sequential phases (Figure 5): *Phase 1--preparing the lattice*. Gp32 rapidly binds and saturates all of the available ssDNA (rapid Gp32F fluorescence increase). *Phase 2--filament growth*. ATP-bound UvsX is loaded by UvsY and gradually displaces Gp32 (slow Gp32F fluorescence decrease). There is a stringent requirement for UvsY and either ATP or ATPγS in this phase, and the rate is optimal when UvsY stoichiometry is 1:1 with respect to UvsX and ssDNA binding sites. *Phase 3--filament collapse*. Depletion of ATP allows Gp32 to slowly re-occupy the ssDNA and drive off UvsX, which is now mainly in the low-affinity ADP/AMP forms [16,48] (slow Gp32F fluorescence increase). This collapse phase is sensitive to the nucleotide substrate/product ratio and does not occur if ATP is regenerated or if ATPγS is substituted. These observations are consistent with a dynamically unstable T4 presynaptic filament. Dynamic instability could take the form of treadmilling as shown in Figure 5, in which UvsX-ssDNA filaments simultaneously grow at an ATP-capped end and contract at an ADP- or AMP-capped end. The vectorial motion would be reinforced by Gp32 which would out-compete UvsX for ssDNA binding sites preferentially at the ADP/AMP-capped filament end.

## Atomic Structure of T4 UvsX Recombinase

A recently solved, high-resolution UvsX crystal structure provides important new information on the mechanism of the T4 recombinase [66]. The crystal was obtained from a truncation mutant UvsX_30-358 _(full-length UvsX = 391 amino acid residues), which lacks the N-terminal protein-protein association domain and the extreme C-terminal region. The crystal has a P6_1 _space group and the asymmetric unit is composed of dimer of identical subunits with a two-fold axis. In the crystal lattice these dimers are arranged as a right-handed helical filament, with one subunit of each dimer forming the filament while the opposite subunit in each dimer decorates the surface of the filament without interacting with its symmetry partners. The dimer interface in the asymmetric unit occludes the ATP binding site, therefore no bound ATP is observed in the structure. The DNA binding loops L1 and L2 of UvsX are disordered as is the case for all RecA family proteins crystallized in the absence of DNA.

As expected, UvsX shares high similarity with *E. coli *RecA protein in overall architecture and protein folding, in spite of the remote sequence homology [67]. Compared to RecA, UvsX contains a larger N-terminal α/β motif, and a smaller C-terminal domain filled with helices and a small three-stranded β-sheet. The α/β ATPase core is highly conserved between UvsX and RecA in terms of structural motifs, locations, and amino acid compositions. The two nucleotide-binding motifs of UvsX, the Walker A and Walker B boxes, are located at similar positions compared to RecA structures. For example, the aromatic ring of Tyr99 in UvsX stacks with the adenine ring of ATP, similar to Tyr103 in RecA [66].

**Figure 6:**
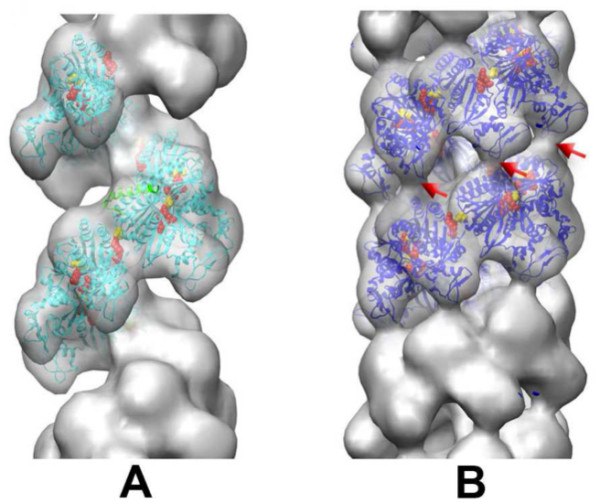
**EM of UvsX recombination filaments (adapted from **[66]**)**. **A**. A reconstruction of the extended 'active' filament (grey) formed in the presence of dsDNA and ATP into which the UvsX crystal structure has been fitted (cyan). The C-terminal helical domain is pointing down towards the large groove. The filament has a rotation per subunit of 58.5° and axial rise per subunit of 16.1 Å. The 28 N-terminal residues of RecA were used to model the missing N-terminal UvsX residues (green ribbons). The positions of three residues in UvsX at the monomer-monomer interface that correspond to those in RecA involved in the ATP hydrolysis are shown as red (K246, R248), and yellow (E92) spheres. **B**. The compressed 'inactive' filament formed in the presence of dsDNA and ADP in which the fitted UvsX structure is shown in dark blue. The filament has a rotation per subunit of 55.7° and axial rise per subunit of 10.8 Å. A bridge of density across the groove, corresponding to an interaction between residues 130-132 of one monomer and residues 285-288 of the other monomer, is shown in red.

Docking of the UvsX structure into models of extended and compressed filament forms reconstituted from EM studies revealed additional details about the active site (Figure 6)[66]. Docking into the high-pitch "active" filament (ADP-AlF_4 _form) indicated that the ATPase site spans the filament interface, as is the case for high-pitch filaments of *E. coli *RecA and *S. cerevisiae *Rad51 [17,68,69]. Conserved residue Glu92 is positioned to activate a water molecule for nucleophilic attack on ATP γ-phosphate. Significantly, residues Lys246' and Arg248' reach across the filament interface and form salt bridges with the phosphates of ATP and with Glu92. These residues are structurally equivalent to the Lys248' and Lys250' bridges and to catalytic residue Glu96 in *E. coli *RecA. The lysine bridges are thought to promote catalysis by stabilizing the transition state during ATP hydrolysis [69]. This strategy is apparently conserved between RecA and UvsX. Interestingly, eukaryotic Rad51 and Dmc1 recombinases lack the entire motif containing the basic bridge residues, and no other basic residues take their places in the Rad51 crystal structures [17,68]. Thus there is a divergence of active site structure and function between the prokaryotic and eukaryotic recombinases, with UvsX more closely aligned to the prokaryotic mechanism.

Docking of the UvsX structure into the low-pitch "inactive" filament (ADP form) indicates that residues Lys246' to Lys254' move by about 4 Å so that the ATP binding site no longer spans the filament interface. These observations indicate that changes in filament pitch observed at different stages of the ATPase cycle are accompanied by extensive remodeling of the active site itself. Overall, the high-resolution structure of UvsX [66] provides exciting new opportunities to investigate its catalytic and allosteric mechanisms.

## Actions of Helicases in DNA Strand Exchange Reactions

The bacteriophage T4 recombination system provided one of the earliest demonstrations that a DNA helicase, Dda protein, can stimulate a recombinase-catalyzed DNA strand exchange reaction [70]. Subsequent work has shown that at least three T4-encoded helicases (Dda, Gp41, and UvsW) are capable of influencing recombination and/or recombination-dependent replication transactions in vitro, and probably in vivo as well. In this section we will focus on the impacts of Dda, Gp41, and UvsW on reconstituted strand exchange reactions *in vitro*.

### Helicase processing of recombination intermediates

After a UvsX-catalyzed homology search and strand pairing, a joint molecule is formed between the invading 3' single-stranded DNA (ssDNA) tail and the homologous double-stranded DNA (dsDNA) template in the form of a displacement-loop (D-loop) (Figure 1). ssDNA regions of the D-loop are potential targets for helicase assembly. Depending on which strand the helicase translocates on, and on the polarity of the helicase, processing of the D-loop could have three different outcomes: extension of the heteroduplex by branch migration, unwinding of the heteroduplex by branch or bubble migration, or conversion of the D-loop into a nascent replication fork. In addition, certain helicases may use their translocase activity to remove presynaptic filaments from ssDNA. It appears likely that all four of these processes occur at some point during T4 DNA metabolism. It has been shown that all three T4 helicases, Dda, Gp41, and UvsW, are capable of catalyzing branch migration *in vitro *[29,70,71]. However, the biological functions of these helicases are distinctive, in spite of the overlapping branch migration activities.

### Dda helicase

Dda is a unique helicase compared to Gp41 and UvsW, since it may regulate recombination both positively and negatively at two different stages: presynaptic filament formation and branch migration. *E. coli *UvrD and yeast Srs2 proteins are two translocases/helicases functioning to remove recombinases from ssDNA and to prevent improper presynaptic filament formation and illegitimate recombination events [72-74]. To date, no T4 helicase has been identified as a direct functional homolog of UvrD or Srs2. Dda may share some properties of these helicases though, since the phenotypes of certain *dda *mutants are consistent with a role in anti-recombination [75], and since Dda inhibits UvsX-mediated homologous strand pairing reactions in vitro [76]. It is speculated that destabilizing UvsX-ssDNA filaments through its translocase activity is one factor contributing to the observed inhibition of homologous pairing. Similarly, Dda might apply this translocation activity to DNA replication by allowing the fork to bypass DNA-bound proteins on the template in vitro [77-79]. If Dda protein does disrupt presynaptic filaments then its mechanism must differ somewhat from Srs2 and UvrD, since the latter two have 3' to 5' polarity while Dda has 5' to 3' polarity [80-82].

The strand exchange assay routinely uses a circular M13 ssDNA and a linearized M13 dsDNA as substrates. The extent of branch migration after initial synapsis can be monitored by the restriction endonuclease digestion pattern of the end-radiolabeled dsDNA [70]. This nicely-designed assay system allowed Kodadek and Alberts to monitor and measure the rate of branch migration of UvsX-catalyzed strand exchange in the presence and absence of Dda. The late addition of Dda after synapsis stimulates the rate of branch migration more than four-fold, from ~15 bp/sec to ~70 bp/sec [70]. Dda was the first helicase documented to stimulate strand exchange reactions by stimulating branch migration, on the premise that it is added late into the reconstituted reaction after synapsis has occurred. Furthermore, the specific protein-protein interaction between Dda and UvsX might be important for this stimulation, since Dda cannot stimulate RecA-catalyzed strand exchange reactions.

*In vitro*, Dda's inhibition of homologous pairing and stimulation of branch migration can be separated by manipulating the addition sequence of Dda into the reconstituted reaction, either simultaneously with UvsX during presynapsis, or after the initiation of synapsis. How Dda balances these opposite activities and cooperates with UvsX *in vivo *remains largely unknown, however. It is observed that UvsX and Dda act synergistically in template switching to allow DNA lesion bypass and to rescue stalled replication forks [4,83]. Furthermore, protein-protein interactions between Dda and the C-terminal domain of Gp32 are required for the DNA replication activities of Dda [37]. These observations suggest that interactions with UvsX or with Gp32 could recruit Dda onto different nucleoprotein intermediates at different stages of the strand exchange process, perhaps regulating the recombination vs. anti-recombination functions of Dda.

### Gp41 helicase and Gp59 helicase loading protein

Gp41, the essential replicative helicase in T4, facilitates both leading strand DNA synthesis catalyzed by the T4 DNA polymerase holoenzyme (Gp43, Gp44/Gp62, and Gp45 proteins), and lagging strand DNA synthesis by recruiting primase Gp61 to reconstitute the T4 primosome [4]. The Gp41 helicase translocates processively on the displaced strand in a 5' → 3' direction, as an asymmetric hexagonal ring on the DNA [84,85].

Gp59 has been classified as a replication mediator protein or helicase loading protein, based on the observation that it is required to load Gp41 onto Gp32-covered ssDNA [4,38,77,86]. Gp59 acts as an adapter protein by interacting with Gp32 at the N-terminus and with Gp41 at the C-terminus [86-88]. It is the key factor for the strand-specific recruitment of primosome onto the displaced strand of a D-loop to covert it into a replication fork during RDR, and to initiate new lagging-strand DNA synthesis during RDR. Gp41 cannot stimulate UvsX-dependent strand exchange unless Gp59 is present, and this stimulation occurs through branch migration [70]. UvsY stimulates homologous pairing, but strongly inhibits branch migration. The branch migration activity can only be recovered by adding Gp41 and Gp59. The protein-protein interaction between Gp59 and the C-terminal acidic domain of Gp32 is important for this rescue [70].

**Figure 7:**
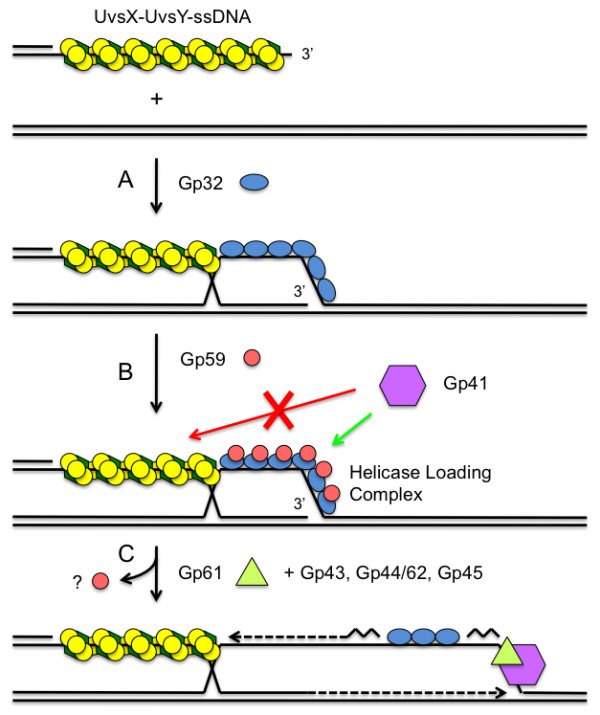
**Conversion of recombination intermediates into replication forks: UvsX/UvsY and Gp59 enforce strand-specific loading of Gp41 helicase onto the displaced strand of a D-loop**. (A) A UvsX-UvsY-ssDNA presynaptic filament invades a homologous dsDNA molecule. Gp32 rapidly sequesters the displaced ssDNA of the D-loop. (B) D-loop ssDNA covered with Gp32 is recognized and bound by Gp59 helicase loading protein, forming a helicase loading complex (HLC). The HLC is shown as an extended structure here for simplicity, but it is actually remodeled into a condensed bead-like structure [37]. Gp59 is excluded from the invading ssDNA, which is saturated with UvsX and UvsY. Therefore Gp41 helicase cannot be loaded onto the invading strand where it would abortively unwind the D-loop (anti-recombination). (C) The HLC loads Gp41 helicase specifically onto the displaced strand of the D-loop. Recruitment of Gp61 primase plus DNA polymerase holoenzyme (Gp43, Gp44/62, Gp45; not shown for simplicity) reconstitutes the semi-conservative recombination-dependent replication machinery. Note that Gp59 inhibits leading strand DNA synthesis until the primosome is reconstituted, so that leading/lagging strand synthesis begins in a coordinated fashion.

Interestingly, the formation and stability of Gp32-ssDNA clusters is a key factor for strand- and structure-specific loading of Gp41 helicase by Gp59. Gp59 targets Gp41 helicase assembly onto Gp32-ssDNA clusters [4,37,38]. The interplay between Gp32 and Gp59 is complicated. The formation of a tripartite Gp59-Gp32-ssDNA complex decreases the stability of Gp32-ssDNA interaction, but Gp32 also helps modulate the strand specificity of Gp59 [4,38]. Gp59-mediated primosome assembly is precluded from ssDNA that is saturated with UvsX and UvsY, but allowed when a few Gp32 clusters interrupt the presynaptic filament. In DNA strand exchange, the invading strand is typically saturated with UvsX and UvsY and therefore resistant to Gp41/Gp59 loading. However, Gp32 rapidly sequesters the displaced strand of the D-loop [19,47], forming a target for Gp41/Gp59. Thus UvsX/UvsY and Gp32/Gp59 enforce strand specific loading of Gp41 onto the displaced strand, where it is poised to catalyze branch migration using its 5' to 3' helicase activity (Figure 7). UvsX/UvsY prevent D-loop resolution (anti-recombination) by Gp41/Gp59 by preventing their assembly on the invading ssDNA strand. An identical partitioning mechanism is used during RDR to ensure primosome assembly on the displaced strand of the D-loop, assuring complete reconstitution of semi-conservative DNA synthesis beginning with a recombination event [4].

In the absence of UvsX and UvsY, the sole presence of excessive amount of Gp32 can produce joint molecules from M13 dsDNA with a 3' single-stranded termini of about 100 nucleotides and a circular M13 ssDNA [89]. The initial binding of Gp32 onto the single-stranded tail is probably sufficient to destabilize the double-stranded helix, starting from the junction point, and to promote spontaneous joint molecule formation. When coupled with Gp59 and Gp41, the polar branch migration mediated by Gp41 can drive the formation of nicked circle, the final product of standard three-strand exchange reactions [89]. This synergism between Gp32 and Gp41/Gp59 is also crucial for extensive strand displacement synthesis by the T4 DNA polymerase holoenzyme [39,90].

### UvsW helicase

UvsW plays a central role in T4 recombination and in the transition from origin to recombination-dependent replication. UvsW mutations cause hypersensitivity to UV and hydroxyurea, and a decreased frequency of recombination [91,92]. UvsW is a 3' to 5' RNA/DNA and DNA/DNA helicase with specificity for branched-DNA substrates such as X-shaped Holliday junctions and Y-shaped replication forks [71,93,94]. It does not unwind linear duplex substrates with either blunt ends or single-stranded tails. Substrate recognition may occur through a small but highly electropositive N-terminal domain and an arginine/aromatic-rich loop, as revealed by its crystal structure [95]. The mutant phenotype and substrate specificity lead to the hypothesis that UvsW might drive branch migration to resolve recombination intermediates during strand invasion and transfer. Indeed, purified UvsW protein can catalyze Holliday junction branch migration through more than 1 kb of DNA sequence, using a plasmid-based Holliday junction-containing substrate [71]. Recent data show that UvsW promotes branch migration in UvsX-catalyzed DNA strand exchange reactions [66]. In the classic three-strand exchange reaction with M13 circular ssDNA and linear dsDNA substrates, UvsW promotes resolution of the branched ssDNA/dsDNA networks formed by UvsX, leading to the robust generation of nicked circular heteroduplex product. Reactions occur in the presence of Gp32 and in either the presence or absence of UvsY. Thus UvsW appears to provide a "missing link" in the biochemistry of T4 recombination, since it can provide physiologically reasonable mechanisms for generating extensive heteroduplex DNA, involving the translocation of either 3- or 4-strand junctions.

In summary, Dda, Gp41, and UvsW are three helicases all capable of stimulating branch migration, but with clearly different biological roles in T4 recombination. Dda may act as a negative regulator of homologous pairing, but may also be used to accelerate branch migration or to couple recombination to bubble migration DNA synthesis [70,75,76,96]. The major role of Gp41/Gp59 in recombination is likely to be the channeling of recombination intermediates into structures that can support RDR, and then launching lagging strand synthesis in the semi-conservative RDR mechanism [4]. UvsW on the other hand optimizes strand exchange and the formation of long heteroduplex DNA [66]. Complex interplays between the three different helicase activities are likely to modulate many aspects of T4 recombination metabolism.

## Conclusions

Studies of the T4 recombination system have provided insights on recombination mechanisms that are highly relevant to HR and HDR processes in cellular organisms including eukaryotes. Work with T4 UvsY protein has helped to define the roles that recombination mediator proteins play in promoting presynaptic filament assembly and in the trafficking of recombination proteins (SSB, RMP, and recombinase) on ssDNA that occurs during the early stages of recombination and homology-directed DNA repair processes. It is clear that the UvsY model for assembly of recombinase filaments on ssDNA covered with ssDNA-binding protein is highly conserved [24], including in human beings where at least three classes of proteins with UvsY-like mediator activity participate in genome stability pathways. These include Rad52, the human Rad51 paralogs Rad51B, Rad51C, Rad51D, Xrcc2, and Xrcc3, and breast cancer susceptibility gene Brca2 [97-100]. Details of T4 presynaptic filament assembly and dynamics, such as ssDNA hand-offs and dynamic instability, suggest mechanisms that may be used by recombination machineries in many organisms to capture recombinagenic ssDNA, perform strand exchange, and pass the intermediates on to other repair enzymes such as the replicative components of HDR pathways.

Recent biochemical and structural studies of UvsX recombinase shed light on its mechanism and relationship to other recombinases of the RecA/Rad51 superfamily. The observation that ssDNA-binding by UvsX allosterically regulates the enzyme's affinity for homologous vs. non-homologous dsDNA at a second site is an important breakthrough [66]. The sensitive fluorescence assay developed for this study represents an excellent opportunity to explore how micro-heterology affects homologous pairing, as well as the similarities and differences between pairing mechanisms used by recombinases from various organisms. The X-ray crystal structure of UvsX and its modeling in EM filament structures shows that UvsX shares the same extended filament structure in its active form as do *E. coli *and yeast filament structures (Gajewski, S., M.R. Webb, V. Galkin, E.H. Egelman, K.N. Kreuzer, and S.W. White: Crystal structure of the phage T4 recombinase UvsX and its functional interation with the T4 SF2 helicase UvsW, unpublished). The observation that UvsX appears to share the lysine bridges found at the active site of *E. coli *RecA-DNA places UvsX mechanistically closer to prokaryotic than to eukaryotic recombinases, at least in this detail. Opportunities for structure-driven mutagenesis and mechanistic studies, as well as for evolutionary studies, of UvsX will surely follow from this important structure.

The T4 field pioneered studies of helicases in recombination, which are now known to be pervasive regulators of recombination and HDR metabolism in all organisms [100]. The biochemistry of T4 helicases demonstrates the diverse ways that these enzymes can influence recombination outcomes, including both positive and negative regulation of homologous pairing and strand exchange. It is noteworthy that T4 encodes threes different helicases on its phage genome that appear to have both unique and overlapping functions in recombination. Of particular relevance is the role of helicases in channeling strand exchange reactions toward the formation of intermediates that can serve as initiators of recombination-dependent DNA replication [4,6,96]. T4 RDR requires either Dda (for bubble-migration DNA synthesis) or Gp41/Gp59 (for semi-conservative DNA synthesis) to initiate replication via a recombination event. The biochemical role of UvsW in the RDR machine remains to be elucidated but is likely to be central given its ability to promote extensive branch migration. The coupling of recombination to replication is fundamental for DNA repair and genome stability in all organisms. Eukaryotic DNA helicases/translocases such as Rad54, Srs2 and others are known to play important roles in processing recombination intermediates, either for regulatory purposes or to facilitate access of downstream DNA replication and repair enzymes to the products of strand exchange [10,11,72-74,100]. The T4 helicases offer an excellent opportunity to study more about the mechanism of recombination/replication coupling, the findings of which will directly inform studies of genome stability mechanisms in cellular organisms including humans.

## Abbreviations

HR: homologous recombination; HDR: homology-directed repair; RDR: recombination-dependent replication; DSB: double-strand break; ssDNA: single-stranded DNA; dsDNA: double-stranded DNA; SSB: single-stranded DNA binding protein; RMP: recombination mediator protein; ATPγS: adenosine 5'-*O*-(3-thio)triphosphate; Gp32F: fluorescein-labeled bacteriophage T4 gene 32 protein (Gp32).

## Competing interests

The authors declare that they have no competing interests.

## Authors' contributions

JL and SM made equal intellectual contributions to this review and participated equally in writing the manuscript.

## References

[B1] Jasin M Homologous repair of DNA damage and tumorigenesis: the BRCA connection Oncogene 2002 21 8981 8993 10.1038/sj.onc.1206176 12483514

[B2] Symington LS Role of RAD52 epistasis group genes in homologous recombination and double-strand break repair Microbiol Mol Biol Rev 2002 66 630 670 10.1128/MMBR.66.4.630-670.2002 12456786 PMC134659

[B3] Pierce AJ Stark JM Araujo FD Moynahan ME Berwick M Jasin M Double-strand breaks and tumorigenesis Trends Cell Biol 2001 11 S52 59 11684443 10.1016/s0962-8924(01)02149-3

[B4] Bleuit JS Xu H Ma Y Wang T Liu J Morrical SW Mediator proteins orchestrate enzyme-ssDNA assembly during T4 recombination-dependent DNA replication and repair Proc Natl Acad Sci USA 2001 98 8298 8305 10.1073/pnas.131007498 11459967 PMC37435

[B5] Mosig G Karam JD Homologous recombination Molecular Biology of Bacteriophage T4 1994 ASM Press, Washington, DC 54 82

[B6] Kreuzer KN Recombination-dependent DNA replication in phage T4 Trends Biochem Sci 2000 25 165 173 10.1016/S0968-0004(00)01559-0 10754548

[B7] Sun H Treco D Szostak JW Extensive 3'-overhanging, single-stranded DNA associated with the meiosis-specific double-strand breaks at the ARG4 recombination initiation site Cell 1991 64 1155 1161 10.1016/0092-8674(91)90270-9 2004421

[B8] Haber JE In vivo biochemistry: physical monitoring of recombination induced by site-specific endonucleases BioEssays 1995 17 609 620 10.1002/bies.950170707 7646483

[B9] Mickelson C Wiberg JS Membrane-associated DNase activity controlled by genes 46 and 47 of bacteriophage T4D and elevated DNase activity associated with the T4 das mutation J Virol 1981 40 65 77 7026800 10.1128/jvi.40.1.65-77.1981 PMC256596

[B10] Li X Heyer WD RAD54 controls access to the invading 3'-OH end after RAD51-mediated DNA strand invasion in homologous recombination in Saccharomyces cerevisiae Nucleic Acids Res 2009 37 638 646 10.1093/nar/gkn980 19074197 PMC2632917

[B11] Macris MA Sung P Multifaceted role of the Saccharomyces cerevisiae Srs2 helicase in homologous recombination regulation Biochem Soc Trans 2005 33 1447 1450 10.1042/BST20051447 16246143

[B12] Bianco PR Tracy RB Kowalczykowski SC DNA strand exchange proteins: a biochemical and physical comparison Front Biosci 1998 3 570 603 10.2741/a304 9632377

[B13] Ando RA Morrical SW Single-stranded DNA binding properties of the UvsX recombinase of bacteriophage T4: binding parameters and effects of nucleotides J Mol Biol 1998 283 785 796 10.1006/jmbi.1998.2124 9790840

[B14] Liu J Bond JP Morrical SW Mechanism of presynaptic filament stabilization by the bacteriophage T4 UvsY recombination mediator protein Biochemistry 2006 45 5493 5502 10.1021/bi0525167 16634631

[B15] Formosa T Alberts BM Purification and characterization of the T4 bacteriophage uvsX protein J Biol Chem 1986 261 6107 6118 2939071

[B16] Farb JN Morrical SW Role of allosteric switch residue histidine 195 in maintaining active-site asymmetry in presynaptic filaments of bacteriophage T4 UvsX recombinase J Mol Biol 2009 385 393 404 10.1016/j.jmb.2008.11.003 19027026 PMC2888526

[B17] Conway AB Lynch TW Zhang Y Fortin GS Fung CW Symington LS Rice PA Crystal structure of a Rad51 filament Nat Struct Mol Biol 2004 11 791 796 10.1038/nsmb795 15235592

[B18] Lauder SD Kowalczykowski SC Asymmetry in the recA protein-DNA filament J Biol Chem 1991 266 5450 5458 1826000

[B19] Kodadek T Wong ML Alberts BM The mechanism of homologous DNA strand exchange catalyzed by the bacteriophage T4 uvsX and gene 32 proteins J Biol Chem 1988 263 9427 9436 2967823

[B20] Riddles PW Lehman IR The formation of plectonemic joints by the recA protein of Escherichia coli. Requirement for ATP hydrolysis J Biol Chem 1985 260 170 173 3880737

[B21] Kowalczykowski SC Krupp RA DNA-strand exchange promoted by RecA protein in the absence of ATP: implications for the mechanism of energy transduction in protein-promoted nucleic acid transactions Proc Natl Acad Sci USA 1995 92 3478 3482 10.1073/pnas.92.8.3478 7724585 PMC42190

[B22] Harris LD Griffith JD UvsY protein of bacteriophage T4 is an accessory protein for in vitro catalysis of strand exchange J Mol Biol 1989 206 19 27 10.1016/0022-2836(89)90520-2 2522995

[B23] Yonesaki T Minagawa T Synergistic action of three recombination gene products of bacteriophage T4, uvsX, uvsY, and gene 32 proteins J Biol Chem 1989 264 7814 7820 2785988

[B24] Beernink HT Morrical SW RMPs: recombination/replication mediator proteins Trends Biochem Sci 1999 24 385 389 10.1016/S0968-0004(99)01451-6 10500302

[B25] Kodadek T Gan DC Stemke-Hale K The phage T4 uvsY recombination protein stabilizes presynaptic filaments J Biol Chem 1989 264 16451 16457 2550444

[B26] Melamede RJ Wallace SS Properties of the nonlethal recombinational repair x and y mutants of bacteriophage T4. II. DNA synthesis J Virol 1977 24 28 40 904025 10.1128/jvi.24.1.28-40.1977 PMC515907

[B27] Melamede RJ Wallace SS Properties of the nonlethal recombinational repair deficient mutants of bacteriophage T4. III. DNA replicative intermediates and T4w Mol Gen Genet 1980 177 501 509 10.1007/BF00271490 6929402

[B28] Kreuzer KN Morrical SW Karam JD Initiation of DNA replication Molecular Biology of Bacteriophage T4 1994 ASM Press, Washington, DC 28 42

[B29] Salinas F Kodadek T Phage T4 homologous strand exchange: a DNA helicase, not the strand transferase, drives polar branch migration Cell 1995 82 111 119 10.1016/0092-8674(95)90057-8 7606776

[B30] Chase JW Williams KR Single-stranded DNA binding proteins required for DNA replication Annu Rev Biochem 1986 55 103 136 10.1146/annurev.bi.55.070186.000535 3527040

[B31] Karpel RL Revzin A T4 bacteriophage gene 32 protein The Biology of nonspecific DNA-protein interactions 1990 CRC Press; Boca Raton, FL 103 130

[B32] Shamoo Y Friedman AM Parsons MR Konigsberg WH Steitz TA Crystal structure of a replication fork single-stranded DNA binding protein (T4 gp32) complexed to DNA Nature 1995 376 362 366 10.1038/376362a0 7630406

[B33] Williams KR Shamoo Y Spicer EK Coleman JE Konigsberg WH Karam JD Correlating structure to function in proteins: T4 Gp32 as a prototype Molecular Biology of Bacteriophage T4 1994 ASM Press, Washington, DC 301 304

[B34] Giedroc DP Khan R Barnhart K Overexpression, purification, and characterization of recombinant T4 gene 32 protein22-301 (g32P-B) J Biol Chem 1990 265 11444 11455 2195020

[B35] Hurley JM Chervitz SA Jarvis TC Singer BS Gold L Assembly of the bacteriophage T4 replication machine requires the acidic carboxy terminus of gene 32 protein J Mol Biol 1993 229 398 418 10.1006/jmbi.1993.1042 8429554

[B36] Jiang H Giedroc D Kodadek T The role of protein-protein interactions in the assembly of the presynaptic filament for T4 homologous recombination J Biol Chem 1993 268 7904 7911 8385125

[B37] Ma Y Wang T Villemain JL Giedroc DP Morrical SW Dual functions of single-stranded DNA-binding protein in helicase loading at the bacteriophage T4 DNA replication fork J Biol Chem 2004 279 19035 19045 10.1074/jbc.M311738200 14871889

[B38] Morrical SW Beernink HT Dash A Hempstead K The gene 59 protein of bacteriophage T4. Characterization of protein-protein interactions with gene 32 protein, the T4 single-stranded DNA binding protein J Biol Chem 1996 271 20198 20207 10.1074/jbc.271.33.20198 8702746

[B39] Xu H Wang Y Bleuit JS Morrical SW Helicase assembly protein Gp59 of bacteriophage T4: fluorescence anisotropy and sedimentation studies of complexes formed with derivatives of Gp32, the phage ssDNA binding protein Biochemistry 2001 40 7651 7661 10.1021/bi010116n 11412119

[B40] Kowalczykowski SC Saenger W Thermodynamic data for protein-nucleic acid interactions 1990 Berlin: Springer-Verlag 244 263 Landolt-Bornstein: Numerical Data and Functional Relationships in Science and Technology (New Series) Group VII: Biophysics, Nucleic Acids 1d

[B41] Kowalczykowski SC Lonberg N Newport JW von Hippel PH Interactions of bacteriophage T4-coded gene 32 protein with nucleic acids. I. Characterization of the binding interactions J Mol Biol 1981 145 75 104 10.1016/0022-2836(81)90335-1 7265204

[B42] Newport JW Lonberg N Kowalczykowski SC von Hippel PH Interactions of bacteriophage T4-coded gene 32 protein with nucleic acids. II. Specificity of binding to DNA and RNA J Mol Biol 1981 145 105 121 10.1016/0022-2836(81)90336-3 7265197

[B43] Pant K Karpel RL Rouzina I Williams MC Salt dependent binding of T4 gene 32 protein to single and double-stranded DNA: single molecule force spectroscopy measurements J Mol Biol 2005 349 317 330 10.1016/j.jmb.2005.03.065 15890198

[B44] Rouzina I Pant K Karpel RL Williams MC Theory of electrostatically regulated binding of T4 gene 32 protein to single- and double-stranded DNA Biophys J 2005 89 1941 1956 10.1529/biophysj.105.063776 15994897 PMC1366697

[B45] Shokri L Rouzina I Williams MC Interaction of bacteriophage T4 and T7 single-stranded DNA-binding proteins with DNA Phys Biol 2009 6 15096 15103 10.1088/1478-3975/6/2/025002 PMC2805082 19571366

[B46] Morrical SW Alberts BM The UvsY protein of bacteriophage T4 modulates recombination-dependent DNA synthesis in vitro J Biol Chem 1990 265 15096 15103 2144282

[B47] Kodadek T The role of the bacteriophage T4 gene 32 protein in homologous pairing J Biol Chem 1990 265 20966 20969 2250001

[B48] Liu J Qian N Morrical SW Dynamics of bacteriophage T4 presynaptic filament assembly from extrinsic fluorescence measurements of Gp32-single-stranded DNA interactions J Biol Chem 2006 281 26308 26319 10.1074/jbc.M604349200 16829679

[B49] Yassa DS Chou KM Morrical SW Characterization of an amino-terminal fragment of the bacteriophage T4 uvsY recombination protein Biochimie 1997 79 275 285 10.1016/S0300-9084(97)83515-8 9258436

[B50] Beernink HT Morrical SW The uvsY recombination protein of bacteriophage T4 forms hexamers in the presence and absence of single-stranded DNA Biochemistry 1998 37 5673 5681 10.1021/bi9800956 9548953

[B51] Xu H Beernink HT Morrical SW DNA-binding properties of T4 UvsY recombination mediator protein: polynucleotide wrapping promotes high-affinity binding to single-stranded DNA Nucleic Acids Res 2010 38 4821 4833 10.1093/nar/gkq219 20371513 PMC2919719

[B52] Sweezy MA Morrical SW Single-stranded DNA binding properties of the uvsY recombination protein of bacteriophage T4 J Mol Biol 1997 266 927 938 10.1006/jmbi.1996.0829 9086271

[B53] Bleuit JS Ma Y Munro J Morrical SW Mutations in a conserved motif inhibit single-stranded DNA binding and recombination mediator activities of bacteriophage T4 UvsY protein J Biol Chem 2004 279 6077 6086 10.1074/jbc.M311557200 14634008

[B54] Pant K Shokri L Karpel RL Morrical SW Williams MC Modulation of T4 gene 32 protein DNA binding activity by the recombination mediator protein UvsY J Mol Biol 2008 380 799 811 10.1016/j.jmb.2008.05.039 18565541 PMC2527458

[B55] Ando RA Morrical SW Relationship between hexamerization and ssDNA binding affinity in the uvsY recombination protein of bacteriophage T4 Biochemistry 1999 38 16589 16598 10.1021/bi991917h 10600121

[B56] McGhee JD Theoretical calculations of the helix-coil transition of DNA in the presence of large, cooperatively binding ligands Biopolymers 1976 15 1345 1375 10.1002/bip.1976.360150710 949539

[B57] Morrical SW Wong ML Alberts BM Amplification of snap-back DNA synthesis reactions by the uvsX recombinase of bacteriophage T4 J Biol Chem 1991 266 14031 14038 1649833

[B58] Farb JN Morrical SW Functional complementation of UvsX and UvsY mutations in the mediation of T4 homologous recombination Nucleic Acids Res 2009 37 2336 2345 10.1093/nar/gkp096 19244311 PMC2673438

[B59] Griffith J Formosa T The uvsX protein of bacteriophage T4 arranges single-stranded and double-stranded DNA into similar helical nucleoprotein filaments J Biol Chem 1985 260 4484 4491 3156858

[B60] Kodadek T Functional interactions between phage T4 and E. coli DNA-binding proteins during the presynapsis phase of homologous recombination Biochem Biophys Res Commun 1990 172 804 810 10.1016/0006-291X(90)90746-A 2241970

[B61] Sweezy MA Morrical SW Biochemical interactions within a ternary complex of the bacteriophage T4 recombination proteins uvsY and gp32 bound to single-stranded DNA Biochemistry 1999 38 936 944 10.1021/bi9817055 9893989

[B62] Hashimoto K Yonesaki T The characterization of a complex of three bacteriophage T4 recombination proteins, uvsX protein, uvsY protein, and gene 32 protein, on single-stranded DNA J Biol Chem 1991 266 4883 4888 2002035

[B63] Echols H Multiple DNA-protein interactions governing high-precision DNA transactions Science 1986 233 1050 1056 10.1126/science.2943018 2943018

[B64] Lohman TM Kowalczykowski SC Kinetics and mechanism of the association of the bacteriophage T4 gene 32 (helix destabilizing) protein with single-stranded nucleic acids. Evidence for protein translocation J Mol Biol 1981 152 67 109 10.1016/0022-2836(81)90096-6 6279865

[B65] Hilario J Amitani I Baskin RJ Kowalczykowski SC Direct imaging of human Rad51 nucleoprotein dynamics on individual DNA molecules Proc Natl Acad Sci USA 2009 106 361 368 10.1073/pnas.0811965106 19122145 PMC2613362

[B66] Gajewski S Webb MR Galkin V Egelman EH Kreuzer KN White SW Crystal Structure of the Phage T4 Recombinase UvsX and Its Functional Interaction with the T4 SF2 Helicase UvsW J Mol Biol 2010 [Epub ahead of print] 21035462 10.1016/j.jmb.2010.10.004 PMC3006652

[B67] Fujisawa H Yonesaki T Minagawa T Sequence of the T4 recombination gene, uvsX, and its comparison with that of the recA gene of Escherichia coli Nucleic Acids Res 1985 13 7473 7481 10.1093/nar/13.20.7473 2932679 PMC322056

[B68] Chen J Villanueva N Rould MA Morrical SW Insights into the mechanism of Rad51 recombinase from the structure and properties of a filament interface mutant Nucleic Acids Res 2010 38 4889 4906 10.1093/nar/gkq209 20371520 PMC2919713

[B69] Chen Z Yang H Pavletich NP Mechanism of homologous recombination from the RecA-ssDNA/dsDNA structures Nature 2008 453 489 484 10.1038/nature06971 18497818

[B70] Kodadek T Alberts BM Stimulation of protein-directed strand exchange by a DNA helicase Nature 1987 326 312 314 10.1038/326312a0 2950327

[B71] Webb MR Plank JL Long DT Hsieh TS Kreuzer KN The phage T4 protein UvsW drives Holliday junction branch migration J Biol Chem 2007 282 34401 34411 10.1074/jbc.M705913200 17823128 PMC2094049

[B72] Krejci L Van Komen S Li Y Villemain J Reddy MS Klein H Ellenberger T Sung P DNA helicase Srs2 disrupts the Rad51 presynaptic filament Nature 2003 423 305 309 10.1038/nature01577 12748644

[B73] Veaute X Delmas S Selva M Jeusset J Le Cam E Matic I Fabre F Petit MA UvrD helicase, unlike Rep helicase, dismantles RecA nucleoprotein filaments in Escherichia coli Embo J 2005 24 180 189 10.1038/sj.emboj.7600485 15565170 PMC544901

[B74] Veaute X Jeusset J Soustelle C Kowalczykowski SC Le Cam E Fabre F The Srs2 helicase prevents recombination by disrupting Rad51 nucleoprotein filaments Nature 2003 423 309 312 10.1038/nature01585 12748645

[B75] Mosig G Recombination and recombination-dependent DNA replication in bacteriophage T4 Annu Rev Genet 1998 32 379 413 10.1146/annurev.genet.32.1.379 9928485

[B76] Kodadek T Inhibition of protein-mediated homologous pairing by a DNA helicase J Biol Chem 1991 266 9712 9718 1851754

[B77] Barry J Alberts B A role for two DNA helicases in the replication of T4 bacteriophage DNA J Biol Chem 1994 269 33063 33068 7806534

[B78] Bedinger P Hochstrasser M Jongeneel CV Alberts BM Properties of the T4 bacteriophage DNA replication apparatus: the T4 dda DNA helicase is required to pass a bound RNA polymerase molecule Cell 1983 34 115 123 10.1016/0092-8674(83)90141-1 6136341

[B79] Gauss P Park K Spencer TE Hacker KJ DNA helicase requirements for DNA replication during bacteriophage T4 infection J Bacteriol 1994 176 1667 1672 8132462 10.1128/jb.176.6.1667-1672.1994 PMC205253

[B80] Jongeneel CV Formosa T Alberts BM Purification and characterization of the bacteriophage T4 dda protein. A DNA helicase that associates with the viral helix-destabilizing protein J Biol Chem 1984 259 12925 12932 6092351

[B81] Nanduri B Byrd AK Eoff RL Tackett AJ Raney KD Pre-steady-state DNA unwinding by bacteriophage T4 Dda helicase reveals a monomeric molecular motor Proc Natl Acad Sci USA 2002 99 14722 14727 10.1073/pnas.232401899 12411580 PMC137486

[B82] Raney KD Benkovic SJ Bacteriophage T4 Dda helicase translocates in a unidirectional fashion on single-stranded DNA J Biol Chem 1995 270 22236 22242 10.1074/jbc.270.38.22236 7673202

[B83] Kadyrov FA Drake JW UvsX recombinase and Dda helicase rescue stalled bacteriophage T4 DNA replication forks in vitro J Biol Chem 2004 279 35735 35740 10.1074/jbc.M403942200 15194689

[B84] Dong F Gogol EP von Hippel PH The phage T4-coded DNA replication helicase (gp41) forms a hexamer upon activation by nucleoside triphosphate J Biol Chem 1995 270 7462 7473 10.1074/jbc.270.27.16302 7706292

[B85] Norcum MT Warrington JA Spiering MM Ishmael FT Trakselis MA Benkovic SJ Architecture of the bacteriophage T4 primosome: electron microscopy studies of helicase (gp41) and primase (gp61) Proc Natl Acad Sci USA 2005 102 3623 3626 10.1073/pnas.0500713102 15738414 PMC553339

[B86] Morrical SW Hempstead K Morrical MD The gene 59 protein of bacteriophage T4 modulates the intrinsic and single-stranded DNA-stimulated ATPase activities of gene 41 protein, the T4 replicative DNA helicase J Biol Chem 1994 269 33069 33081 7806535

[B87] Delagoutte E von Hippel PH Mechanistic studies of the T4 DNA (gp41) replication helicase: functional interactions of the C-terminal Tails of the helicase subunits with the T4 (gp59) helicase loader protein J Mol Biol 2005 347 257 275 10.1016/j.jmb.2005.01.036 15740739

[B88] Ishmael FT Alley SC Benkovic SJ Assembly of the bacteriophage T4 helicase: architecture and stoichiometry of the gp41-gp59 complex J Biol Chem 2002 277 20555 20562 10.1074/jbc.M111951200 11927580

[B89] Kong D Nossal NG Richardson CC Role of the bacteriophage T7 and T4 single-stranded DNA-binding proteins in the formation of joint molecules and DNA helicase-catalyzed polar branch migration J Biol Chem 1997 272 8380 8387 10.1074/jbc.272.13.8380 9079662

[B90] Nossal NG Karam JD The bacteriophage T4 replication fork Molecular Biology of Bacteriophage T4 1994 ASM Press, Washington, DC 43 53

[B91] Derr LK Drake JW Isolation and genetic characterization of new uvsW alleles of bacteriophage T4 Mol Gen Genet 1990 222 257 264 10.1007/BF00633826 2274029

[B92] Derr LK Kreuzer KN Expression and function of the uvsW gene of bacteriophage T4 J Mol Biol 1990 214 643 656 10.1016/0022-2836(90)90283-R 2388264

[B93] Carles-Kinch K George JW Kreuzer KN Bacteriophage T4 UvsW protein is a helicase involved in recombination, repair and the regulation of DNA replication origins Embo J 1997 16 4142 4151 10.1093/emboj/16.13.4142 9233823 PMC1170037

[B94] Dudas KC Kreuzer KN UvsW protein regulates bacteriophage T4 origin-dependent replication by unwinding R-loops Mol Cell Biol 2001 21 2706 2715 10.1128/MCB.21.8.2706-2715.2001 11283250 PMC86901

[B95] Sickmier EA Kreuzer KN White SW The crystal structure of the UvsW helicase from bacteriophage T4 Structure 2004 12 583 592 10.1016/j.str.2004.02.016 15062081

[B96] Formosa T Alberts BM DNA synthesis dependent on genetic recombination: characterization of a reaction catalyzed by purified bacteriophage T4 proteins Cell 1986 47 793 806 10.1016/0092-8674(86)90522-2 3022939

[B97] Liu J Doty T Gibson B Heyer WD Human BRCA2 protein promotes RAD51 filament formation on RPA-covered single-stranded DNA Nat Struct Mol Biol 2010 17 1260 1262 10.1038/nsmb.1904 20729859 PMC2952495

[B98] Jensen RB Carreira A Kowalczykowski SC Purified human BRCA2 stimulates RAD51-mediated recombination Nature 2010 467 678 683 10.1038/nature09399 20729832 PMC2952063

[B99] San Filippo J Sung P Klein H Mechanism of eukaryotic homologous recombination Annu Rev Biochem 2008 77 229 257 10.1146/annurev.biochem.77.061306.125255 18275380

[B100] Sung P Klein H Mechanism of homologous recombination: mediators and helicases take on regulatory functions Nat Rev Mol Cell Biol 2006 7 739 750 10.1038/nrm2008 16926856

